# Geographical disparities in infant mortality in the rural areas of China: a descriptive study, 2010–2018

**DOI:** 10.1186/s12887-022-03332-z

**Published:** 2022-05-12

**Authors:** Xue Yu, Yanping Wang, Leni Kang, Lei Miao, Xiaowei Song, Xuemei Ran, Jun Zhu, Juan Liang, Qi Li, Li Dai, Xiaohong Li, Chunhua He, Mingrong Li

**Affiliations:** 1 National Office for Maternal and Child Health Surveillance of China, West China Second University Hospital, Sichuan University, Chengdu, Sichuan China; 2 Department of gynaecology and obstetrics, Maternal and Child healthcare hospital of Dujiangyan, Chengdu, Sichuan, China; 3 Department of pediatrics, Hanyuan people’s Hospital, Hanyuan, Sichuan Yaan, China; 4 Key Laboratory of Birth Defects and Related Diseases of Women and Children of the Ministry of Education, West China Second University Hospital, Sichuan University, Chengdu, Sichuan, China

**Keywords:** Infant mortality rate, Leading causes, Geographical disparities, Rural China

## Abstract

**Background:**

The infant mortality rate (IMR) is considered a basic measure of public health for countries around the world. The specific aim of our study was to provide an updated description of infant mortality rate among different regions in rural China, and assess the trends and causes of the IMR geographical disparities.

**Methods:**

Data were collected from China’s Under-5 Child Mortality Surveillance System(U5CMSS). The annual number of deaths and causes of death were adjusted using a 3-year moving average underreporting rate based on annual national data quality control results. The average annual decline rate (AADR) and the relative risk (RR) of the IMR and cause-specific infant mortality were calculated by Poisson regression and the Cochran–Mantel–Haenszel method. Data analysis was completed by SAS software.

**Results:**

There was an apparent decrease in infant mortality in rural China from 2010 to 2018, at the AADR of 11.0% (95%CI 9.6–12.4), 11.2% (95%CI 10.3–12.1) and 6.6% (95%CI 6.0–7.3) in the eastern, central and western rural areas, respectively. The IMR was highest in the western rural area, followed by the central and eastern rural areas. Compared with the eastern rural area, the RR of infant mortality in the central rural area remained at 1.4–1.6 and increased from 2.4 (95%CI 2.3–2.6) in 2010–2012 to 3.1 (95% CI 2.9–3.4) in 2016–2018 in the western rural area. Pneumonia, preterm birth /LBW and birth asphyxia were the leading causes of infant deaths in the western rural area. Mortality rates of these three causes fell significantly in 2010–2018 but contributed to a higher proportion of deaths in the western rural area than in the central and western rural ares.

**Conclusions:**

Our study indicated that the infant mortality rate dropped significantly from 2010 to 2018, however, geographical disparities of IMR in rural China are still persist. Therefore, there is an urgent need for public health programmes and policy interventions for infants in western rural China.

## Introduction

The infant mortality rate (IMR) is considered a basic measure of public health for countries around the world, reflecting the apparent association between the causes of infant mortality and other factors that are likely to influence the health status of the whole population such as their economic development, general living conditions, social well-being, rates of illness and the quality of the environment [1, 2]. Economic development, together with the expansion of health care services, has led to significant reductions in child mortality worldwide [3]. Globally, the infant mortality rate decreased from 65 deaths per 1000 live births to 29 deaths per 1000 live births in 1990–2018, and annual infant deaths declined from 8.7 million in 1990 to 4.0 million in 2018 [4]. However, improvements in national child mortality are likely to be accompanied by increases in inequalities and disparities in child survival across countries. Children from the poorest households are five times more likely to die before their fifth birthday than children from wealthier households [5] Children are particularly vulnerable to the impact of social disadvantages and inequities, which are evident from birth and have a profound effect on health throughout their lifespan [6]. Under the slogan “leaving no one behind”, the 2030 Agenda of the United Nations has emphasized the importance of equity in achieving the Sustainable Development Goals (SDGs) [4].

China represents a unique example of success in achieving the Millennium Development Goal 4(MDG4) target of reducing the under-5 mortality rate by two-thirds between 1990 and 2015 [7]. China has made great strides in improving child survival in the past decades, with a reduction in the infant mortality rate from 32.2 deaths per 1000 live births in 2000 to 6.1 deaths per 1000 live births in 2018 [8]. Despite encouraging advancements in the reduction of infant mortality, progress has been uneven across and within countries [9]. China, like many other countries, also has large inequalities in infant mortality rates according to socioeconomic development and geographic region [10]. National figures from 2018 published by the Office for National Statistics showed that the IMR was 7.3 deaths per 1000 live births in rural areas, which was two times that of urban areas [8]. In addition, regional differences are even more pronounced between the remote rural area in the west and the developed rural area in the east [11]. Children living in poor rural areas have significantly worse health outcomes than their counterparts in wealthier, urban areas [12]. Therefore, improving the health of children in rural China requires more sustained efforts by the government.

Effective programs to reduce the infant mortality rate in rural areas require accurate epidemiological characteristics of infant deaths. One article investigated the trends and causes of the IMR geographical disparities from 1996 to 2008 in rural China [10]. However, with changes in economics, the environment, medical techniques and health policies, infant mortality rates have undergone great changes in China. It is important to provide an up-to-date description according to the most recent available data. The specific aim of our study was to provide an updated description of infant mortality rates among different regions in rural China and assess the trends and causes of the IMR geographical disparities.

## Methods

### Data source

The data were obtained from the China’s Under-5 Child Mortality Surveillance System (U5CMSS), a population-based monitoring system used to collect important statistics on child mortality levels and causes of death. There are approximately 47.1 million individuals across 334 representative sites in the 31 provinces of mainland China [7]. According to the criteria from the National Development and Reform Commission of China, these 31 provinces were divided into eastern, central, and western regions, and each region was further stratified into urban and rural areas according to the criteria used in the National Health Services Survey and the administrative division codes published by the National Bureau of Statistics. The causes of death were classified according to the International Classification of Diseases-10 (ICD-10). The standard procedures for data collection, reporting, auditing and quality control in the surveillance network have been described elsewhere [13]. In this study, U5CMSS data are available annually for the period from 2010 to 2018.

### Assessment of causes of death

Doctors from the township hospital or community health service centre performed household surveys for each death in their respective areas, described the symptoms before the death in detail on the child death registration card, and assessed the causes of death. The cause of death was determined from death certificates in the case of children who died in the hospital, hospital-reported medical diagnoses in the case of children who received health care before their death, or verbal autopsies when medical records were not available. All causes of deaths were confirmed by pediatricians at maternal and child health institutes at the county, municipal and provincial levels. Furthermore, the neonatal death review program and the under-5 child death review program were implemented at most surveillance sites before nationwide implementation in 2009. These programs mainly focused on the assessment of causes of death.

### Statistical analysis

In this study, U5CMSS data are available for each year in the period from 2010 to 2018. Infant mortality was defined as the number of deaths per 1000 live births, and the rate of cause-specific infant mortality was calculated as the number of cause-related deaths per 100,000 live births. The infant mortality and cause-specific infant mortality were adjusted by the 3-year moving average under-reporting rate based on annual national data quality control results [13]. The average annual decline rate (AADR) of the IMR and its 95% confidence interval (95%CI) were calculated by Poisson regression. The relative risk (RR) of the IMR, cause-specific infant mortality and its 95%CI among different regions in rural China were measured using the Cochran-Mantel-Haenszel method. Trends in the IMR, cause-specific infant mortality and the proportions of infants who sought medical services before death among the three regions in rural China were assessed for significance using the χ^2^ trend test. An infant’s diagnostic level composition ratio was calculated based on the reported child death report card, excluding children who died from accidental injuries [7].

All statistical analyses in this study were performed using SAS software.

## Results

**Fig. 1:**
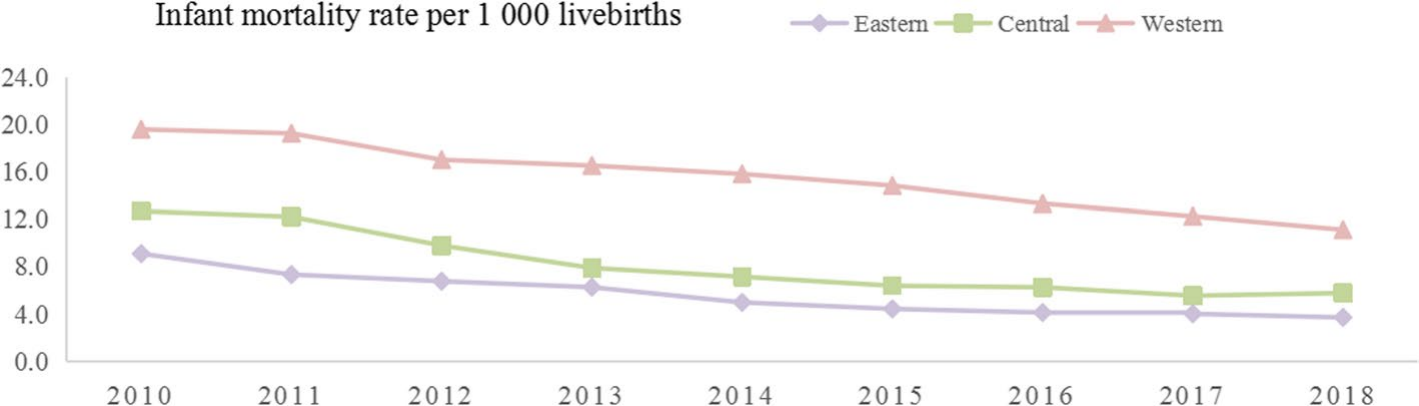
The infant mortality rate among the regions in rural China during 2010–2018

A total of 2,066,576 live births and 21,926 infant deaths were registered in the U5CMSS from 2010 to 2018. Of the 21,926 infant deaths, 11.2% occurred in the eastern rural area, 30.9% occurred in the central rural area and 57.9% occurred in the western rural area. The infant mortality declined substantially within each region of rural China between 2010 and 2018, which decreased from 9.1, 12.7 and 19.6 deaths per 1000 livebirths in 2010 to 3.7, 5.8 and 11.1 deaths per 1000 live births in 2018, respectively, resulting in AADRs of 11.0% (95%CI 9.6–12.4), 11.2% (95%CI 10.3–12.1) and 6.6% (95%CI 6.0–7.3), in the eastern, central and western rural areas, respectively, (*p* < 0.001) (Fig. 1).

During the study period, there were large geographical differences in infant mortality rate (IMR), neonatal mortality rate (NMR) and post-neonatal mortality rate (PMR) in rural China. The rates were the highest in the western rural area, followed by the central and eastern rural areas. Compared with the eastern rural area, the RR of IMR remained at 1.4–1.6 in the central rural area during 2010–2018, and increased from 2.4 (95%CI 2.3–2.6) in 2010–2012 to 3.1 (95%CI 2.9–3.4) in 2016–2018 in the western rural area. Using the eastern rural area as the reference, the RR of NMR decreased from 1.6 (95%CI 1.5–1.8) in 2010–2012 to 1.4 (95%CI 1.2–1.5) in 2016–2018 in the central rural area, and remained at 2.4–2.8 in the western rural area. The RR of PMR in central and eastern rural areas increased from 1.3 (95%CI 1.1–1.4) and 2.4 (95%CI 2.2–2.7) in 2010–2012 to 1.8 (95%CI 1.5–2.1) and 3.9 (95%CI 3.3–4.5), relative to the eastern rural area, respectively (Table 1).

**Table 1:** Risks of infant mortality, neonatal mortality and post-neonatal mortality among regions of rural China during 2010-2018

Location		2010-2012			2013-2015		2016-2018			
	death	mortality rate^a^	RR^b^	death	mortality rate^a^	RR^b^ (95%CI)	death	mortality rate^a^	RR^b^(95%CI)	
			(95%CI)							
Infant										
Eastern	1084	7.7	1	759	5.2	1	605	3.9	1	
Central	3374	11.6	1.5(1.4-1.6)	1963	7.2	1.4(1.3-1.5)	1436	5.9	1.5(1.4-1.6)	
Western	5078	18.6	2.4(2.3-2.6)	4403	15.7	3.0(2.8-3.3)	3225	12.3	3.1(2.9-3.4)	
Neonatal										
Eastern	666	4.7	1	503	3.4	1	401	2.6	1	
Central	2274	7.8	1.6(1.5-1.8)	1360	5.0	1.4(1.3-1.6)	878	3.6	1.4(1.2-1.5)	
Western	3127	11.5	2.4(2.2-2.6)	2737	9.8	2.8(2.6-3.1)	1927	7.3	2.8(2.5-3.1)	
Post neonatal										
Eastern	418	3.0	1	255	1.7	1	197	1.3	1	
Central	1100	3.8	1.3(1.1-1.4)	603	2.2	1.3(1.1-1.5)	558	2.3	1.8(1.5-2.1)	
Western	1951	7.2	2.4(2.2-2.7)	1665	5.9	3.4(3.0-3.9)	1298	4.9	3.9(3.3-4.5)	

^a^mortality rate was calculated as the number of deaths per 1000 live births

^b^RRof infant mortality, neonatal mortality and post-neonatal mortality in the central or western rural areas relative to the eastern rural areas.

In our study, the main diseases that caused infant deaths were congenital anomalies, preterm birth /LBW, injuries, birth asphyxia, pneumonia, and diarrhoea, which accounted for 85% of the total infant death in rural China. In 2016–2018, the top three causes of infant deaths were congenital anomalies (29.0%), preterm birth /LBW (14.8%) and pneumonia (11.9%) in the eastern rural area, congenital anomalies (26.0%), preterm birth /LBW (16.5%) and birth asphyxia (13.1%) in the central rural area, and pneumonia (20.8%), preterm birth /LBW (17.3%) and congenital anomalies (16.1%) in the western rural area. In the eastern rural area, birth asphyxia was the leading cause of infant deaths in 2010–2012, accounting for 15.6% of all deaths, and the contribution of birth asphyxia dropped to 10.2%, making it the 4th leading cause in 2016–2018. In the western rural area, the proportions of infant deaths due to congenital abnormalities increased from 13.4% in 2010–2012 to 16.1% in 2016–2018, making it the third leading cause in 2016–2018, the contribution of pneumonia was stable in this period, ranging between 19.3 and 20.8% during the study period (Table 2).

**Table 2:** The number of cause-specific infant deaths, the cause-specific mortailty rate and the proportion(%) in regions of rural China during 2010-2018

	2010-2012			2013-2015			2016-2018			2010-2018		
	Deaths	mortality^a^	Proportion	Deaths	mortality^a^	Proportion	Deaths	mortality^a^	Proportion	Deaths	Proportion	AADR^b^ (95%CI)
Eastern												
Congenital abnormalities	314	223.1	29.0%	181	123.8	23.8%	175	113.9	29.0%	670	27.4%	11.8%(9.1, 14.4)
Prematurity/LBW	258	183.2	23.8%	180	123.3	23.7%	89	58.2	14.8%	527	21.5%	16.4%(13.5, 19.3)
Injuries	105	74.3	9.6%	66	44.9	8.6%	57	37.1	9.4%	227	9.3%	11.6%(6.9, 16.0)
Birth asphyxia	169	120.0	15.6%	126	85.9	16.5%	61	39.9	10.2%	356	14.5%	16.2%(12.6, 19.7)
Pneumonia	130	92.3	12.0%	68	46.6	9.0%	72	46.9	11.9%	270	11.0%	11.5%(7.2, 15.6)
Diarrhoea	20	14.0	1.8%	13	8.6	1.7%	3	2.2	0.6%	36	1.5%	26.5%(14.5, 16.7)
Central												
Congenital abnormalities	783	268.0	23.2%	518	188.9	26.5%	373	152.2	26.0%	1674	24.7%	11.8%(9.1, 14.4)
Prematurity/LBW	711	243.6	21.1%	327	119.2	16.6%	236	96.3	16.5%	1274	18.8%	15.2%(13.2, 17.2)
Injuries	356	122.1	10.5%	241	87.9	12.3%	167	67.9	11.6%	764	11.3%	10.0%(7.3, 12.5)
Birth asphyxia	579	198.3	17.2%	309	112.6	15.7%	188	76.6	13.1%	1076	15.9%	15.0%(12.8, 17.1)
Pneumonia	399	136.5	11.8%	236	86.2	12.0%	166	67.7	11.6%	801	11.8%	11.7%(9.1, 14.0)
Diarrhoea	54	18.5	1.6%	24	8.9	1.2%	8	3.4	0.6%	87	1.3%	18.8%(11.0, 25.8)
Western												
Congenital abnormalities	680	249.4	13.4%	640	228.7	14.5%	518	197.5	16.1%	1838	14.5%	4.2%(2.5, 5.9)
Prematurity/LBW	1114	408.2	21.9%	950	339.6	21.6%	556	212.1	17.3%	2620	20.6%	9.1%(7.7, 10.5)
Injuries	418	153.1	8.2%	359	128.4	8.2%	240	91.6	7.50%	1017	8.0%	10.0%(7.3, 12.5)
Birth asphyxia	841	308.2	16.6%	706	252.5	16.0%	418	159.5	13.0%	1965	15.5%	10.0%(9.4, 11.6)
Pneumonia	1016	372.5	20.0%	849	303.6	19.3%	671	255.7	20.8%	2536	20.0%	6.6%(5.0, 8.1)
Diarrhoea	288	105.5	5.7%	233	83.2	5.3%	156	59.5	4.8%	677	5.3%	18.8%(11.0, 25.8)

^a^cause-specific mortality was calculated as the number of specific-caused deaths per 10 000 live births

^b^AADR the average annual decline rate of the cause-specific infant mortality during 2010-2018

The main cause-specific infant mortality within each region of rural China have significantly decreased from 2010 to 2018 (*p* < 0.001). The main cause of infant deaths in the eastern and western rural areas decreased at AADRs greater than 10%, and there was no significant difference in AADRs between the eastern and central rural areas. In the western rural area, the reduction in mortality due to congenital abnormalities 4.2% (95%CI 2.5–5.9), prematurity/LBW 9.1% (95%CI 7.7–10.5) and pneumonia 6.6% (95%CI 5.0–8.1) was particularly slow (Table 2).

Geographical inequality is also manifested in specific causes of infant mortality rates in rural China. Using the eastern rural area as the reference, RRs of the cause-specific infant mortality rate ranged between 1.3 (95%CI 1.1–1.6) and 1.9 (95%CI 1.4–2.6) in the central rural area and between 1.7 (95%CI 1.5–2.1) and 26.9 (95%CI 9.7–95.5) in the western rural area in 2016–2018. The gap in infant mortality rate between the eastern and central rural areas widened for congenital abnormalities, preterm birth /LBW and birth asphyxia, and between the eastern and western rural areas widened for congenital abnormalities, preterm birth /LBW and pneumonia in 2010–2018. The RRs of the main cause-specific infant mortality rates showed no significant variation between the eastern and central rural areas in 2010–2018. The relative risks of infant mortality rates due to preterm birth /LBW, birth asphyxia and pneumonia between the eastern and western rural areas significantly increased over the study period (Table 3).

**Table 3:** Risks of cause-specific infant mortality among regions of rural China during 2010-2018

Cause of death	Cause-specific mortality^a^			RR^b^ (95%CI)		
	Eastern	Central	Western	Eastern	Central	Western
2010-2012						
Congenital abnormalities	223.1	268.0	249.4	1	1.2(1.1,1.4)	1.1(1.0, 1.3)
Prematurity/LBW	183.2	243.6	408.2	1	1.3(1.2,1.5)	2.2(1.9, 2.5)
Injuries	74.3	122.1	153.1	1	1.6(1.3, 2.0)	2.1(1.7, 2.5)
Birth asphyxia	120.0	198.3	308.2	1	1.7(1.4, 2.0)	2.6(2.2, 3.0)
Pneumonia	92.3	136.5	372.5	1	1.5(1.2, 1.8)	4.0(3.4, 4.8)
Diarrhoea	14.0	18.5	105.5	1	1.3(0.8, 2.2)	7.6(4.7, 11.7)
2013-2015						
Congenital abnormalities	123.8	188.9	228.7	1	1.5(1.3,1.8)	1.8(1.6,2.2)
Prematurity/LBW	123.3	119.2	339.6	1	1.0(0.8,1.2)	2.8(2.4, 3.2)
Injuries	44.9	87.9	128.4	1	2.0(1.5, 2.6)	2.9(2.2, 3.7)
Birth asphyxia	85.9	112.6	252.5	1	1.3(1.1, 1.6)	2.9(2.4, 3.5)
Pneumonia	46.6	86.2	303.6	1	1.9(1.4, 2.4)	6.5(5.1, 8.3)
Diarrhoea	8.6	8.9	83.2	1	1.0(0.5, 1.9)	9.7(5.4, 16.4)
2016-2018						
Congenital abnormalities	113.9	152.2	197.5	1	1.3(1.1,1.6)	1.7(1.5, 2.1)
Prematurity/LBW	58.2	96.3	212.1	1	1.7(1.3, 2.1)	3.6(2.9, 4.6)
Injuries	37.1	67.9	91.6	1	1.8(1.4, 2.5)	2.5(1.8, 3.3)
Birth asphyxia	39.9	76.6	159.5	1	1.9(1.4, 2.6)	4.0(3.1, 5.3)
Pneumonia	46.9	67.7	255.7	1	1.4(1.1, 1.9)	5.5(4.3, 7.0)
Diarrhoea	2.2	3.4	59.5	1	1.5(0.4, 6.3)	26.9(9.7, 95.5)

^a^cause-specific mortality was calculated as the number of specific-caused deaths per 10 000 live births

^b^RR of cause-specific mortality in the central or western rural areas relative to the eastern rural areas

**Fig. 2:**
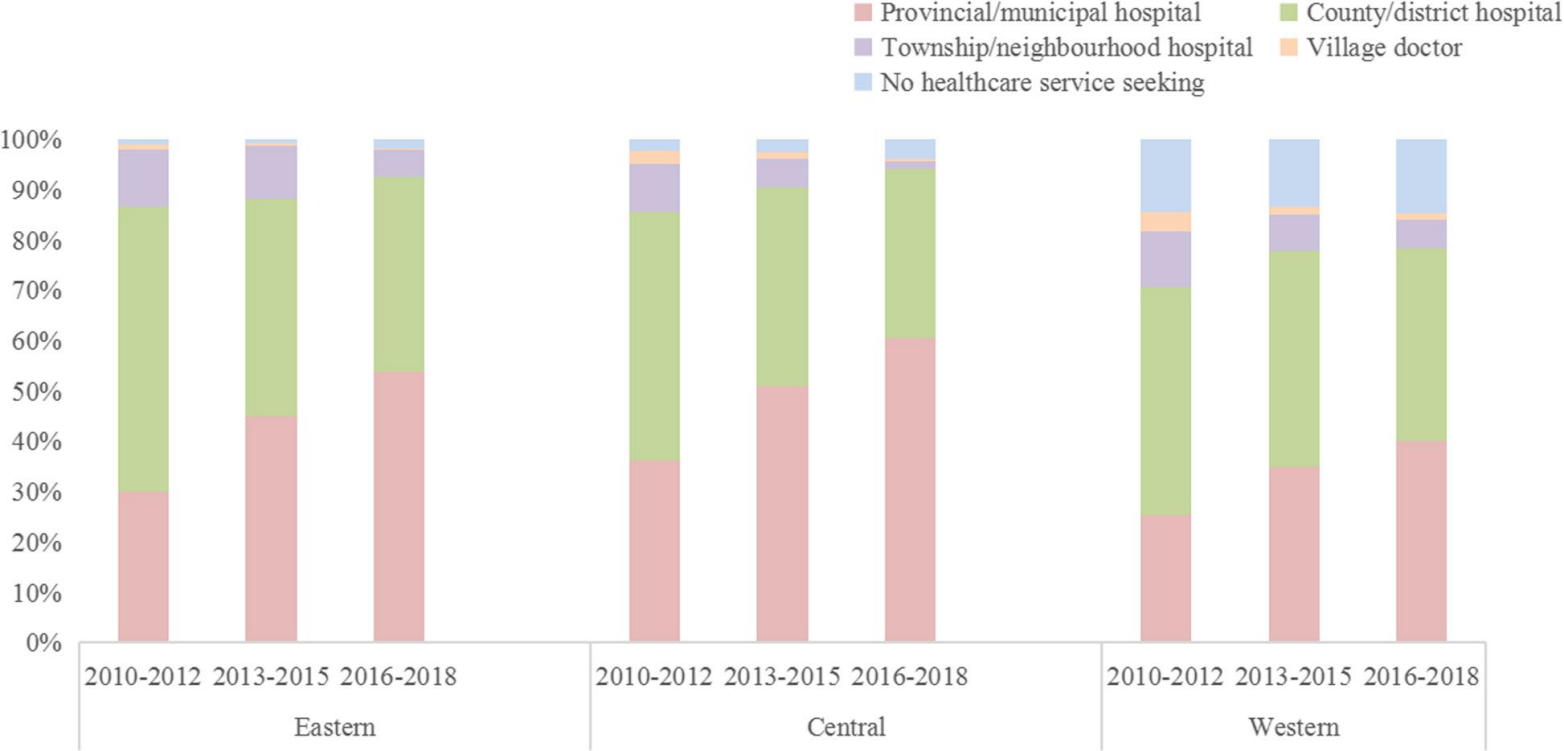
The proportion of pre–mortality health care service among the regions in rural China during 2010–2018

We found that parents’ willingness to seek medical treatment significantly increased in rural areas during 2010–2018. However, the geographical disparities in premortality health care service still existed in rural areas. In 2010–2018, 92.5, 94.0 and 78.3% of infants sought medical treatment at the county/district level or higher hospitals in the eastern, central and western rural areas, respectively. The growth rate of children’s visits to provincial/municipal hospital was highest in the central rural area (from 36.1% in 2010–2012 to 60.6% in 2016–2018), followed by the eastern rural area (from 30.0% in 2010–2012 to 53.7% in 2016–2018) and western rural area (from 25.4% in 2010–2012 to 40.0% in 2016–2018). The proportion of children who did not receive any medical attention still showed a large difference (eastern:1.2%, central: 2.9%, western: 14.2%) (Fig. 2).

## Discussion

In the period of 2010–2018, the infant mortality has decreased significantly in rural China. It has achieved an impressive average annual rate of decline of infant mortality rate of 11.0, 11.2 and 6.6% in the eastern, central and western rural areas, respectively. These decreases may be attributed to rapid economic growth in rural China. In 2018, the per capita disposable income was ¥14,617 (about the US $2234) in rural China, with an increase of 748% over the past decade [14]. The proportion of the annual spending on healthcare per capita in rural China has also increased from 7.4% in 2010 to 10.2% in 2018 [15]. Implementation of major policies and programs on maternal and child survival and health could be another important contributor [16]. For example, the Safe Motherhood Initiative and the national Program to Reduce Maternal Mortality and Eliminate Neonatal Tetanus implemented in 2012 has increased institutional delivery and eliminated neonatal tetanus [17], the New Rural Cooperative Medical System implemented in 2003 has reduced the rural residents’ expenses on hospitalization and improved the utilization of inpatient services among children [18], the ‘Healthy China 2030 Planning Outline’ promulgated in 2016 has raised awareness of injury prevention among children and parents and reduced theunintentional injury mortality [19]. In addition, the construction of the pediatric institution, and implement neonatal asphyxia recovery training programme has also contributed to the decline in IMR [20].

However, there are still large regional differences in IMRs in rural China in 2010–2018. Relative to the eastern rural area, the RR of IMR in the central rural area remained at 1.4–1.6, while the RR in the western rural area increased from 2.4 to 3.1 during the study period. Socioeconomic factors are repeatedly considered to be strong determinants of population health, especially for infants [21]. For example, the IMR in the USA varies by the urbanization level of maternal residence, being the lowest in large urban counties and highest in rural areas [22], and the IMR was 5.9 per 1000 live births in the most deprived areas and 2.6 per 1000 live births in the least deprived areas in England [23]. The average annual growth rate of the per capita GDP in the eastern, central and western regions of rural China increased form ¥23,658.4, ¥12,563.9 and ¥13,919.0 in 2013 to ¥36,298.2, ¥23,798.2 and ¥21,935.8 in 2018, respectively [24], and the average annual growth rate of the per capita GDP in the eastern region was 1.7 times that of the western region in 2018 [24]. Inequity in health infrastructure and services may be another major factor in the regional disparities in infant mortality. In our study, Table 2 shows that the proportion of infants who sought medical treatment at the county/district level before death in remote areas was significantly lower than that in the other two regions. In China, most of high-quality health resources are concentrated in hospitals, especially tertiary hospitals, of 46.7% are located in the eastern area [25]. A national health care survey in 2018 showed that the number of health care workers was 7.2 and 7.9 per 1000 individuals in the eastern and central regions, while it was 6.9 per 1000 individuals in the western region [26]. In addition, the harsh natural environments and difficult access to convenient transportation methods might also contribute to a lack of health services in the western rural area [27]. Parental education has also been linked to lasting improvements in child health and life expectancy through direct and indirect effects mediated by other determinants of health, such as socioeconomic status and living conditions [28–30]. The education level of individuals in the eastern region is higher than that of individuals in the central and western regions [31], and the proportion of the population with education and higher education in the eastern regions was 38.6 and 46.5%, respectively, while it was 26.5 and 23.5% in the western region, respectively [15].

Almost half of all infant deaths (57.6%) occurred in the least developed western rural area during the study period. The leading cause of infant deaths was pneumonia, which is similar to that in other low-income regions [32]. Meanwhile, the proportion of preterm birth /LBW and birth asphyxia in the western rural area are also relatively high. In addition, from 2010 to 2018, the IMR due to preterm birth /LBW, pneumonia and birth asphyxia significantly decreased in the western rural area but was still higher than in the eastern and central rural areas. As preterm birth /LBW, pneumonia and birth asphyxia are preventable and treatable with high coverage of effective interventions, it is important to control these deaths in the western rural area and reduce the geographic disparities during the post-2018 period. The biggest target to reduce infant mortality is preterm birth /LBW in the western rural area, which is similar with the poorer states of India [33]. Prematurity is strongly linked to largely modifiable maternal and prenatal factors, such as antenatal care, education, nutrition and other factors [34]. Therefore, the primary and secondary prevention of preterm birth /LBW, such as antenatal corticosteroids, magnesium sulfate and antibiotic prophylaxis, should receive more attention from the government and the general public in the western rural area [35]. It is recognized as a ‘disease of the poor’, as pneumonia is more likely to affect children living in conditions of poverty and starvation, or who reside in remote regions [36]. In our study, the IMR due to pneumonia in the western rural area was four times higher than that in the eastern rural area. Actions to reduce the incidence of pneumonia, the introduction of polyvalent pneumococcal, Hib and rotavirus vaccines, and the use of oral rehydration salts and zinc, which are still not a standard practice in western rural China [37, 38]. Speeding up the incorporation of these vaccines for pneumonia into the free immunization program should be an effective measure in the western rural area [39]. In addition, the local governments need to strengthen the construction of the Regional Neonatal Transport Network (RNTN), the Neonatal Intensive Care Unit (NICU) and emergency medical care centre [20, 40, 41], increasing the number of well-trained pediatricians and midwives is of great significance to improving infant health in western China, and local health departments can regularly organize multilevel health training programs [20].

There are some limitations in this study. First, we did not have data on factors related to infant deaths, such as household income, maternal education level and age, which prevented us from exploring the relationship between these factors and the infant mortality rate. Second, we did not collect data on health care services for the whole population. Thus, our analysis was based on the health care provided to infants before their death and our results regarding health care accessibility were biased.

## Conclusion

With the joint efforts of the country’s rapid economic growth and policies and programs to eliminate poverty and improve child health, the infant mortality rate dropped significantly in rural China from 2010 to 2018. However, great geographical disparities in IMR still persist in rural China. Almost half of infant deaths occurred in the least developed western rural area during the study period, and IMR in the western rural area was two to three times higher than that in the eastern rural area. Pneumonia and preterm birth /LBW were the main causes of infant deaths in the western rural area and were more prevalent than those in other areas. Therefore, expansion of public health policies, capital investment, health education, and access to medical resources should be a priority for reducing infant mortality rate in the western rural area.

## Data Availability

This study used data from the U5CMSS. This system is co-established by the National Health and Family Planning Commission of the People Republic of China and Sichuan University and finally owned by the National Health and Family Planning Commission of the People Republic of China. The researchers did not obtain consent to publicly share data. The identified data set is available upon request to interested researchers. For data requests, please contact the Department of Science and Technology of West China Second University Hospital, Sichuan University, at: fu2yuankjb@163.com. This department is in charge of all the programs in the hospital, including the data management. One staff from the department monitors this email.

## Abbreviation

IMR: Infant mortality rate
NMR: Neonatal mortality rate
PMR: Post-neonatal mortality rate
U5CMSS: China’s Under-5 Child Mortality Surveillance System
AADR: Average annual decline rate
RR: Relative risk
LBW: Low birth weight
ICD-10: Classification of Diseases-10
SDGs: Sustainable Development Goals
MDG4: Millennium Development Goal 4
RNTN: The Regional Neonatal Transport Network
NICU: Neonatal Intensive Care Unit
